# Health insurance literacy and the associated factors in Iran: A national-scale study

**DOI:** 10.1016/j.hpopen.2025.100150

**Published:** 2025-10-25

**Authors:** Zahra Asadi-Piri, Foroozan Abdollahi-pour, Namam Ali Azadi, Rajabali Daroudi, Ebrahim Jaafaripooyan

**Affiliations:** a National Center for Health Insurance Research,Tehran, Iran; b National Center for Health Insurance Research, Tehran, Iran; c Biostatistics Department, School of Public Health, Iran University of Medical Sciences, Hemmat Expressway, Next to Milad Tower, University of Medical Sciences, Tehran, Iran; d Department of Health Management, Policy and Economics, School of Public Health, Tehran University of Medical Sciences, Tehran, Iran; e PhD Candidate in Health Services Management, Department of Health Management, Policy and Economics, School of Public Health, Tehran University of Medical Sciences, Tehran, Iran

**Keywords:** Health Insurance Literacy, Health Insurance Policy-making, Iran

## Abstract

**Background:**

Health insurance literacy (HIL) plays a vital role in individuals’ ability to understand, select, and use health insurance services. Despite its significance, limited national-level research has been conducted in Iran.

**Methods:**

A cross-sectional study was conducted from November 2024 to February 2025 with 1,625 beneficiaries of the Iranian Health Insurance Organization (IHIO). A validated researcher-developed questionnaire was used assessing the insured groups’ knowledge, attitudes, information-seeking, utilization of services, digital and financial literacy and numeracy. Data were analyzed using t-tests, ANOVA, and multiple linear regression.

**Results:**

The mean HIL score was50.85 ± 20.41. Approximately 25.3% of the participants had low and 35.6% moderate HIL. Significant differences were observed across provinces. The variables associated with the HIL included gender, marital status, occupation, education level, insurance fund type, income, and age. The higher education, employment, and older age were positively associated with the higher HIL scores.

**Conclusion:**

HIL allegedly plays an essential role in the informed healthcare decision-making and equitable service utilization. Disparities seemingly exist across the demographic and regional groups in terms of HIL. Therefore, the tailored educational programs, workplace training, and clearer insurance communication tools are recommended to improve the HIL and reduce inequalities in Iran’s healthcare system.

What is already known about the topic?

Health insurance literacy (HIL), a subdomain of health literacy, is essential for informed decision-making and effective use of insurance benefits. Globally, inadequate HIL is linked to the delays in care, increased healthcare costs, and poor service utilization. In Iran, research on HIL has been limited to local studies or tool development, with no national-scale investigation addressing regional disparities or sociodemographic determinants.

What does this study add to the literature?

This is the first nationally representative study of HIL in Iran, covering over 1,625 individuals from diverse insurance funds and provinces. It revealed significant inequalities in HIL related to age, gender, education, employment, income, and geographic location—providing empirical evidence to inform the national health policy making.

What are the policy implications?

Findings underscore the need for targeted and equity-oriented HIL interventions. Policymakers should implement accessible public education campaigns, digital advisory platforms, and workplace-based training to improve HIL among vulnerable groups. Such strategies can support more informed insurance use and promote equitable access to healthcare.

**Background:**

Health literacy, defined as “the capacity of individuals to obtain, interpret, and understand basic health information and services needed to make appropriate health decisions,” has garnered increasing attention in recent years [1]. The World Health Organization (WHO) recognizes health literacy as a major determinant of health in contemporary societies. It encompasses a set of cognitive and social skills that promote motivation and ability to access, understand, and use information to maintain and improve health [2].

## Highlights

• National survey shows moderate Health Insurance Literacy score.
• HIL varies significantly by gender,age, education, job, and income.
• Men, older adults, and highly educated groups report higher HIL levels.
• Some less-developed provinces outperform urban areas in HIL scores.
• Findings call for targeted, equity-focused HIL education interventions.

Health literacy plays a crucial role in enabling individuals to make informed decisions in complex healthcare systems and, along with socioeconomic and contextual factors, significantly influences access to preventive and therapeutic services [[3], [4], [5]]. Low health literacy has been directly and indirectly linked to a wide range of adverse health outcomes and poor health status in various populations [6]. Numerous studies have demonstrated a close association between health literacy and socioeconomic indicators such as race, education level, age, and disability status. In general populations, a substantial proportion of adults (estimated at 23 %–37 %) have inadequate or marginal health literacy [[7], [8], [9], [10]].

Health insurance literacy (HIL), as a specialized subset of health literacy, refers to individuals’ abilities to access, comprehend, evaluate, and use information related to health insurance [11]. This emerging concept also intersects with financial literacy, forming a unique domain requiring both health and financial decision-making skills [12].

Effective use of health insurance requires specific competencies, such as understanding insurance structures and benefits, estimating treatment costs, and navigating financial responsibilities [13,14]. Challenges surrounding HIL have intensified in recent years due to widespread reforms in health insurance systems and frequent changes in plan structures. Newly insured individuals, especially those with limited prior experience, are particularly vulnerable to low HIL[15,16]. Moreover, rapid changes in insurance offerings and rising out-of-pocket costs have heightened public concern regarding financial risks and out-of-network care [15]. As a result, many individuals delay necessary care due to confusion and uncertainty, which can lead to poorer health outcomes and increased healthcare costs [17].

Having adequate HIL is essential for assessing the financial and health implications of insurance plans. Without this knowledge, consumers may delay or forgo needed care [18,19]. Studies show that individuals with lower HIL struggle more in selecting appropriate insurance plans, while those with higher HIL are more capable of choosing plans aligned with their needs and values [20,21]. Timely access to appropriate insurance coverage plays a vital role in preventing and managing diseases [[22], [23], [24]].

Thus, HIL represents a distinct dimension of health literacy and related self-efficacy, with a notable influence on health service utilization. It emphasizes practical understanding of insurance coverage, benefits, cost-sharing mechanisms, and other critical details essential for optimal use of healthcare services [25]. In light of continuous changes in health insurance and care systems, examining HIL is vital to understanding how, when, and why individuals seek care.

Recent research indicates that, unlike health literacy, the HIL has been less frequently investigated, with significant disparities observed across different demographic groups. For example, a national survey in the United States found that young adults aged 18–29, individuals with lower education levels, and the uninsured generally lack adequate knowledge of basic insurance concepts [26]. Furthermore, several studies have shown that many individuals struggle to understand insurance terms such as premiums, deductibles, and copayments [17]. In one survey, only 14 % of insured respondents could correctly answer four questions related to insurance structure, such as deductible amount, copay requirements, public insurance options, and out-of-pocket limits [27].

However, the existing literature on the HIL is primarily based on the U.S. insurance model, which focuses on individual competencies in selecting and evaluating insurance plans. In contrast, in Iran’s healthcare system—marked by challenges such as inflation and limited oversight of healthcare markets[28]—the skills necessary for navigating insurance benefits and making informed consumer decisions are of heightened importance. In recent years, Iranian insurance organizations have increasingly emphasized on the education and empowerment of their members[29].

In Iran, the healthcare system is based on a mix of public and private providers, with a universal insurance coverage largely delivered through the Iranian Health Insurance Organization (IHIO). The broader health insurance ecosystem in Iran includes several main actors: The Social Security Organization (SSO), which primarily covers employees in the formal sector and their dependents; the Armed Forces Medical Services Insurance Organization, which covers military personnel and their families; and the IHIO, which serves as the largest public insurer. Smaller and specialized funds also exist for specific population groups.

The IHIO was formally established under the Universal Health Insurance Act enacted in 1934 and later reorganized through the Fifth Development Plan and Article 31 of this law, with its statute approved by the Council of Ministers in 1953. Its purpose was to expand the scope and quality of health insurance, achieve universal and equitable coverage of health services, and reduce the share of out-of-pocket health expenditures[30]. According to the Sixth Development Plan, beginning in 1953 this organization was separated from the Ministry of Cooperatives, Labour, and Social Welfare and became an independent body under the Ministry of Health and Medical Education, with its statute subsequently revised[31].

Under its statute, the organization is mandated to take measures, provide facilities, and implement basic universal health insurance for all Iranian citizens in accordance with laws and regulations. Its services are delivered through several insurance funds, each designed for specific population groups: the Rural and Nomadic Insurance Fund, Government Employees Fund, Universal Health Insurance Fund, and the “Other Groups” Fund, which covers war veterans, seminary students, international students, clients of welfare and relief organizations, foreign nationals, prisoners, and unidentified individuals[32]. According to the official report of the IHIO in 2025, the total number of insured individuals across these funds exceeded 42 million [33]. These funds aim to provide equitable insurance coverage across all regions and demographic segments.

Previous research in both Iran and other countries has highlighted the potential influence of sociodemographic and economic factors on health insurance literacy (HIL). Variables such as age, gender, educational attainment, employment status, income level, and type of insurance coverage have been identified as key determinants shaping individuals’ ability to understand and effectively use insurance services[[34], [35], [36], [37]]. For example, studies conducted in Tehran and Ilam indicated that participants with higher levels of education and stable employment demonstrated greater HIL, while younger adults and those from lower-income households consistently reported lower literacy levels. Despite these findings, existing evidence remains limited to local or small-scale studies, leaving considerable uncertainty regarding how these factors operate at the national level in Iran. To address this gap, the present study aims to provide a comprehensive assessment of HIL and its associated sociodemographic and economic factors using a nationally representative sample of beneficiaries of the Iranian Health Insurance Organization (IHIO).

## 1 Methods

This cross-sectional study was conducted between November 2024 and February 2025 among IHIO beneficiaries. Stratified random sampling was applied across provinces and insurance fund types to ensure representativeness. The sampling frame included all IHIO beneficiaries recorded in the national insurance database. Strata were defined by [1] province of residence (covering all 31 provinces of Iran) and (2) type of insurance fund (Universal, Rural and Nomadic, Government Employees, and Other Groups). Within each stratum, potential participants were randomly selected and invited.

**Fig. 1:**
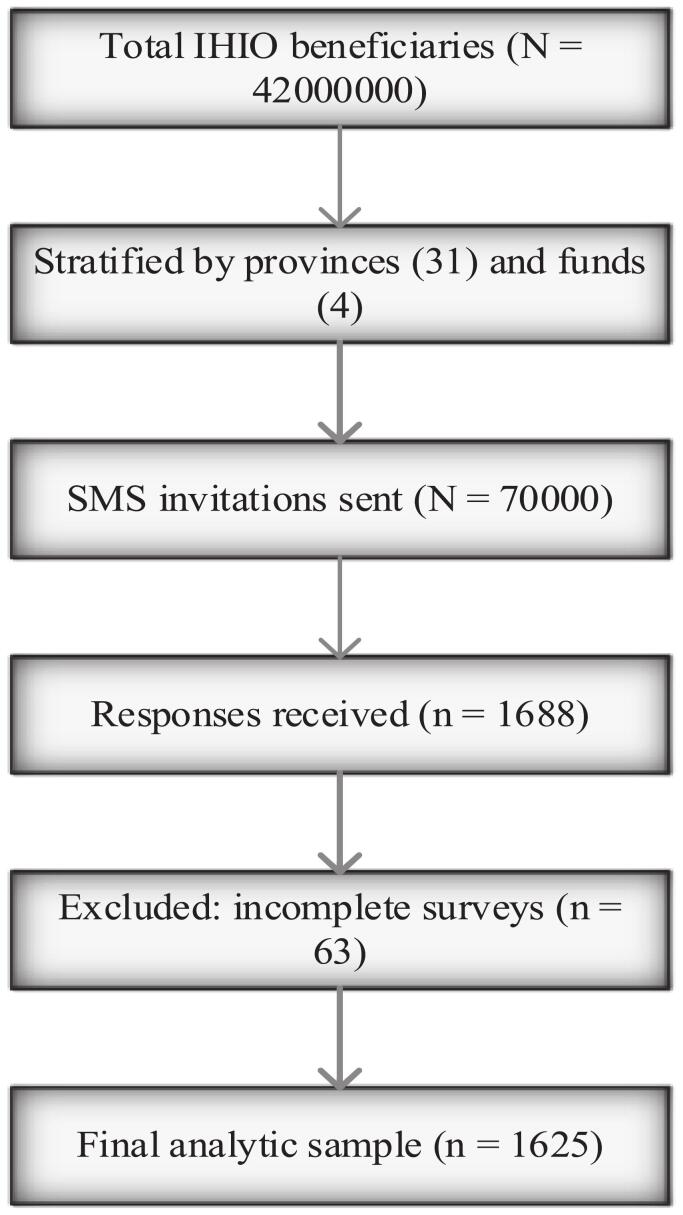
Sampling process.

A total of 70,000 invitation first and follow-up messages were distributed, and 1,625 participants aged ≥ 18 years completed the survey**,** resulting in a response rate of 100 % (Fig. 1). The final analytic sample was proportionally distributed across provinces and insurance funds, closely mirroring the actual IHIO beneficiary population structure, which supports the representativeness of the findings.

### 1.1 Data collection

Data were collected using an online questionnaire distributed via SMS invitations sent through the IHIO. Each insured individual received a text message containing a secure link to the survey. This method was selected to maximize the accessibility, as SMS communication is widely used across Iran—including in rural and nomadic areas—and does not require advanced digital literacy or prior familiarity with the online platforms.

### 1.2 Instrument

The HIL was measured using a researcher-developed and validated tool, drawing mainly upon an existing study [34] and other similar studies ([[38], [39]]). Initially, the instrument was developed with 44 items across five conceptual domains based on the existing literature and previously validated questionnaires. Following expert validation (CVI = 0.82, CVR = 0.63), the instrument was administered and underwent factor analysis. As a result of this empirical evaluation, three items were removed due to the low or cross factor loadings, and the structure was refined from five to seven domains, resulting in the final version consisting of 41 items across seven domains. The instrument demonstrated high internal consistency (overall Cronbach’s α = 0.94). The domains were as follows:

• **Knowledge (10 items):** assesses familiarity with basic insurance concepts such as premiums, deductibles, and coverage limits. Example: *“I know what is meant by deductible in my health insurance policy.”* (Cronbach’s α = 0.89)
• **Attitudes (5 items):** evaluates perceptions toward insurance benefits and trust in insurers. Example: *“I believe having health insurance reduces my financial risk in case of illness.”* (Cronbach’s α = 0.82)
• **Information-seeking behavior (6 items):** examines strategies used to obtain insurance-related information. Example: *“I search the IHIO website to find information about coverage.”* (Cronbach’s α = 0.80)
• **Utilization of insurance services (10 items):** captures experience in accessing and using insurance benefits. Example: *“I have used my insurance card to cover hospitalization expenses.”* (Cronbach’s α = 0.88)
• **Digital literacy** (3 **items**): assesses the beneficiary's awareness of digital platforms (websites and applications) and the practical ability to install and utilize these platforms for accessing, managing, and tracking their health insurance services and information. (Cronbach’s α = 0.70)
• **Financial literacy** (4 **items**): assesses the beneficiary's awareness of personal financial responsibilities and their ability to interpret medical bills and monitor payment accuracy. (Cronbach’s α = 0.72)
• **Numeracy (3 items):** assesses ability to calculate costs and payments. Example: *“I can calculate the amount of coinsurance I must pay when receiving services.”* (Cronbach’s α = 0.87)

Responses were scored on a three-point Likert scale (0 = No, 1 = Somewhat, 2 = Yes). Raw scores (0–82) were normalized to a 0–100 scale. Based on these standardized scores, the HIL was classified into five levels: very low, low, moderate, high, and very high literacy.

### 1.3 Exploratory factor analysis (EFA)

To examine the underlying latent structure of the 41-item HIL instrument, an Exploratory Factor Analysis (EFA) was conducted using Principal Component Analysis (PCA) as the extraction method, followed by Varimax rotation to achieve a simpler and more interpretable factor structure.

Prior to EFA, the data's suitability for factor analysis was assessed. The Kaiser-Meyer-Olkin (KMO) measure of sampling adequacy was 0.951, which is strong (KMO > 0.9), confirming that the data were highly suitable for factor extraction. Bartlett's Test of Sphericity was statistically significant (χ2(861) = 33451.720, p < 0.001), indicating that the correlation matrix was not an identity matrix and that sufficient correlation existed between the variables to proceed with EFA.

Based on the Kaiser criterion (eigenvalues ≥ 1.0), the analysis identified seven factors, collectively accounting for 58.42 % of the total variance in the HIL instrument's items. The initial eigenvalues for these factors ranged from 13.253 (Factor 1) to 1.035 (Factor 7). After Varimax rotation, the cumulative variance explained by the 7-factor solution remained at 58.42 %, with the rotated components explaining a minimum of 5.77 % (Factor 7) and a maximum of 13.14 % (Factor 1) of the variance. Items were retained within a factor if they had a factor loading of 0.4 or greater and showed no significant cross-loadings. This threshold is commonly used in social science research, particularly for larger samples, to ensure that the item contributes meaningfully to the factor structure [40].

### 1.4 Statistical analysis

Descriptive statistics (Mean ± SD, frequencies) summarized participant characteristics. Independent t-tests and ANOVA assessed subgroup differences. Normality of continuous variables was tested using the Shapiro–Wilk test. Variables with *p* < 0.2 in univariate analysis were entered stepwise into a multiple linear regression model. Associations between demographic factors and HIL were analyzed using linear regression. Model assumptions (residual normality, multicollinearity using VIF, and model fit indices such as F-statistic) were tested to ensure robustness of findings.

## 2 Results

### 2.1 Participant characteristics

The mean age of the participants was 52.63 years with a SD of 13.23. Approximately, 71 % were male. Employees Fund represented the largest group with almost 51.69 %, and 62.46 % participants held university-level education. In addition, around 89 % resided in urban areas, and 92 % were Primary Insured (Table 1)**.**

**Table 1:** Univariate Analysis of the Relationship between Total HIL Score and Its Dimensions with Demographic Variables.

**Variable**		**N (%)**	**Conceptual Knowledge (Mean ± SD) (%)**	**P-value**	**Attitudes towards Health Insurance (Mean ± SD) (%)**	**P-value**	**Insurance Information Seeking (Mean ± SD) (%)**	**P-value**	**Utilization of Health Insurance (Mean ± SD) (%)**	**P-value**
**Gender**	Female	476 (29.3 %)	54.48 ± 26.47	<0.0001	76.47 ± 24.94	0.01	56.65 ± 25.67	0.4	30.69 ± 0.25.93	0.07
	Male	1149 (70.7 %)	51.28 ± 27.41		79.59 ± 23.95		57.67 ± 27.60		33.23 ± 26.12	
**Marital Status**	Single	340 (20.9 %)	42.35 ± 26.51	<0.0001	73.83 ± 26.28	<0.0001	52.59 ± 26.33	<0.0001	29.46 ± 26.32	0.01
	Married	1283 (79 %)	55.12 ± 26.66		79.98 ± 23.57		58.65 ± 28.12		33.32 ± 25.98	
**Occupation**	Unemployed	305 (18.8 %)	38.00 ± 25.33	<0.0001	77.04 ± 25.70	<0.0001	49.83 ± 27.00	<0.0001	27.35 ± 23.90	<0.0001
	Housewife	178 (11 %)	36.74 ± 23.64		78.59 ± 23.30		55.24 ± 25.80		28.84 ± 24.86	
	Employee	458 (28.2 %)	54.90 ± 25.77		75.04 ± 26.72		53.93 ± 27.77		32.96 ± 26.39	
	Self-employed	684 (42.1 %)	61.33 ± 25.14		81.87 ± 21.67		62.25 ± 25.97		35.39 ± 26.71	
**Education**	Non-academic	610 (37.5 %)	39.02 ± 25.60	<0.0001	79.85 ± 23.01	0.13	53.64 ± 27.40	<0.0001	28.71 ± 24.64	<0.0001
	Academic	1015 (62.4 %)	60.51 ± 24.70		77.98 ± 25.01		59.61 ± 26.59		34.77 ± 26.67	
**Insurance Fund Type**	Iranian Universal Health Insurance Fund	334 (20.55 %)	41.03 ± 25.45	<0.0001	81.5 ± 21.7	0.09	51.52 ± 27.76	<0.0001	29.87 ± 0.24.33	0.02
	Rural and Nomadic Insurance Fund	139 (8.55 %)	37.01 ± 26.59		79.2 ± 23.6		51.49 ± 28.00		30.25 ± 26.17	
	Employees’ Fund	840(51.69 %)	63.93 ± 23.22		78 ± 24.4		60.62 ± 26.00		34.33 ± 26.32	
	other	308(18.95 %)	40.62 ± 25.15		77.6 ± 27.7		57.50 ± 27.24		31.31 ± 26.94	
**Residence**	Rural	181 (11.1 %)	43.50 ± 28.57	<0.0001	77.84 ± 25.65	0.62	57.09 ± 28.56	0.88	34.11 ± 26.62	0.37
	Urban	1444 (88.9 %)	53.56 ± 26.72		78.78 ± 24.12		57.41 ± 26.86		32.28 ± 26.02	
**Income**	>30 M Toman	30 (1.8 %)	67.00 ± 23.02	<0.0001	52.77 ± 28.77	0.05	52.77 ± 28.89	<0.0001	35.68 ± 27.66	0.01
	20–30 M Toman	113 (7 %)	69.46 ± 22.86		83.62 ± 21.4**2**		64.67 ± 27.84		40.26 ± 28.83	
	10–20 M Toman	811 (49.9 %)	59.71 ± 24.84		78.18 ± 24.4**1**		59.41 ± 26.07		33.30 ± 25.57	
	<10 M Toman	671 (41.3 %)	40.15 ± 25.53		78.76 ± 24.3**0**		53.88 ± 27.54		30.05 ± 25.87	
**Insurance Role**	Primary Insured	1487 (91.5 %)	53.48 ± 27.08	<0.0001	79.01 ± 24.05	0.06	57.52 ± 27.13	0.45	32.63 ± 24.06	0.46
	Dependent	138 (8.5 %)	41.34 ± 24.88		75.07 ± 26.58		55.73 ± 26.19		30.91 ± 26.57	
**Special Diseases Coverage**	Yes	84 (5.2 %)	47.85 ± 26.48	0.11	80.83 ± 20.60	0.40	56.84 ± 27.65	0.85	35.71 ± 24.57	0.24
	No	1541 (94.8 %)	52.69 ± 27.13		78.56 ± 24.47		57.40 ± 27.02		32.31 ± 26.16	
**Incurable disease**	Yes	591(36.36 %)	53.52 ± 26.91	0.02	82.04 ± 21.57	<0.0001	56.44 ± 26.33	0.37	42.10 ± 23.14	0.85
	No	1033(63.63 %)	50.44 ± 27.92		76.75 ± 25.44		54.92 ± 26.75		41.13 ± 24.62	

**Variable**		**N (%)**	**Digital literacy (Mean ± SD) (%)**	**P-value**	**Financial literacy (Mean ± SD) (%)**	**P-value**	**Numeracy skills related to insurance (Mean ± SD) (%)**	**P-value**	**Total HIL (Mean ± SD) (%)**	**P-value**
**Gender**	Female	476 (29.3%)	44.50±30.58	<0.0001	65.57±25.50	0.04	36.76 ± 35.09	0.001	48.90± 19.52	0.02
	Male	1149 (70.7%)	50.95±32.24		62.76±25.97		29.80 ± 32.91		51.36± 20.79	
**Marital Status**	Single	340 (20.9%)	46.51±31.79	0.09	57.81±25.84	<0.0001	31.27 ± 32.84	0.7	45.49 ± 20.54	<0.0001
	Married	1283 (79%)	49.76±31.93		65.11±25.67		31.99 ± 33.94		52.03 ± 20.22	
**Occupation**	Unemployed	305 (18.8%)	**45.57**±**30.99**	<0.0001	**54.95**±25.79	<0.0001	28.30 ± 32.47	<0.0001	43.02 ± 19.17	<0.0001
	Housewife	178 (11%)	**41.85**±31.30		**62.42**±25.61		38.20 ± 34.69		45.44 ± 19.48	
	Employee	458 (28.2%)	**49.34**±32.23		**64.72**±26.33		33.80 ± 34.67		51.03 ± 20.41	
	Self-employed	684 (42.1%)	**52.31**±31.82		**66.99**±24.78		30.45 ± 33.08		55.11 ± 20.01	
**Education**	Non-academic	610 (37.5%)	43.08±30.93	<0.0001	57.99±26.08	<0.0001	28.36 ± 32.34	<0.001	44.85 ± 19.83	<0.0001
	Academic	1015 (62.4%)	52.66±31.94		66.96±25.15		33.94± 34.34		54.15 ± 20.02	
**Insurance Fund Type**	Iranian Universal Health Insurance Fund	334 (20.55%)	52.64±33.03	0.001	61.56±25.29	<0.0001	31.18±32.72	0.89	46.77±19.59	<0.0001
	Rural and Nomadic Insurance Fund	139 (8.55%)	42.92±31.46		56.65±26.63		33.57±33.39		44.72±21.27	
	Employees’ Fund	840(51.69%)	50.31±31.65		50.31±31.65		31.62±33.65		54.83±19.54	
	other	312(9.04%)	44.60±30.77		44.60±30.77		32.37±35.12		46.25±21.04	
**Residence**	Rural	181 (11.1%)	47.32±31.72	0.43	58.28±27.03	0.004	33.42± 33.31	0.50	48.10 ± 22.26	0.07
	Urban	1444 (88.9%)	49.28±31.92		64.24±25.65		31.64 ± 33.76		50.95± 20.20	
**Income**	>30M Toman	30 (1.8%)	**50.55**±**33.47**	<0.0001	**72.91**±18.30	<0.0001	46.11 ± 36.79	0.02	55.71. ± 18.81	<0.0001
	20-30M Toman	113 (7%)	**61.06**±31.85		**70.04**±26.55		30.04 ± 32.76		60.09 ± 20.54	
	10-20M Toman	811 (49.9%)	**49.48**±31.40		**65.87**±25.28		43.8 ± 26.9		52.91 ± 19.67	
	<10M Toman	671 (41.3%)	**46.47**±32.00		**59.36**±26.09		32.86 ± 34.29		46.13 ± 20.41	
**Insurance Role**	Primary Insured	1487 (91.5%)	49.36±32.05	0.22	63.95±25.80	0.05	31.94 ± 33.59	0.1	51.04 ± 20.43	0.01
	Dependent	138 (8.5%)	59.55±26.27		59.55±26.27		35.62 ± 34.77		46.27 ± 20.25	
**Special Diseases Coverage**	Yes	84 (5.2%)	51.98±32.14	0.38	64.50±28.39	0.74	28.57 ± 30.56	0.36	50.57 ± 20.54	0.97
	No	1541 (94.8%)	48.90±31.88		63.53±25.73		32.02 ± 33.86		50.64 ± 20.45	
**Incurable Disease**	Yes	591(36.36%)	49.94±32.77	0.40	65.30±24.64	0.04	28.20±31.93	0.001	51.51±19.68	0.19
	No	1033(63.63%)	48.56±31.38		62.60±26.94		33.93±34.52		50.14±20.86	

### 2.2 HIL level

The mean HIL score was 50.85 (out of 100) with a standard deviation of 20.41 (Table 2). Among the various dimensions, Attitudes Toward Health Insurance had the highest average score (78.68 ± 24.29), while Numeracy Skills Related to Insurance had the lowest (31.84 ± 33.70).

**Table 2:** Total Score of HIL (%) and the Dimensions.

**Dimensions**	**Mean ± SD**	**95 % CI**
**Conceptual Knowledge**	52.44 ± 27.11	(95 % CI:51.23 − 53.76)
**Attitudes toward Health Insurance**	78.68 ± 24.29	(95 % CI:77.54 − 79.84)
**Health Insurance Information Seeking**	57.37 ± 27.04	(95 % CI:55.98 − 58.67)
**Utilization of Health Insurance**	32.23 ± 26.14	(95 % CI:31.01 − 33.53)
**Digital Literacy**	49.06 ± 31.89	(95 % CI:47.50 − 50.60)
**Financial Literacy**	63.14 ± 26.18	(95 % CI:61.83 − 64.37)
**Numeracy Skills related to Insurance**	31.84 ± 33.70	(95 % CI:30.19 − 33.41)
**Total HIL**	50.85 ± 20.41	(95 % CI:49.84 − 51.79)

**Fig. 2:**
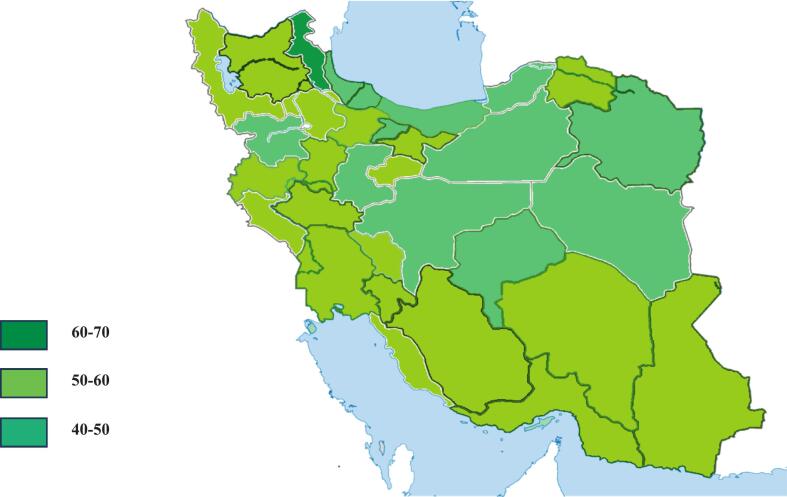
Distribution of Average Health Insurance Literacy Across the Provinces of Iran.

The distribution of HIL levels showed that 25.3 % of participants had a low level, 35.6 % a moderate level and only 9.5 % had a *very high* level (Table 3). A provincial comparison revealed statistically significant differences in the literacy scores. Ardabil province had the highest mean score (66.09 ± 20.95), while Kurdistan province recorded the lowest (42.74 ± 20.85) (Table 4)(Fig. 2). HIL scores were significantly associated with gender (P = 0.07), marital status (P = 0.01), occupation (P < 0.0001), educational level (P < 0.0001), insurance fund type (P = 0.02) and Income(P = 0.01) (Table 1).

**Table 3:** HIL Levels among the Insured Population.

**Dimensions**	**Very Low (%)**	**Low (%)**	**Moderate (%)**	**High (%)**	**Very High (%)**
**Conceptual Knowledge**	16.7	19.4	26.8	21.2	15.9
**Attitudes Toward Health Insurance**	3.8	7.0	15.0	20.6	53.6
**Health Insurance Information Seeking**	10.2	14	32.4	19.2	24.1
**Utilization of Health Insurance**	46.1	24.2	15.3	7.9	6.5
**Digital literacy**	25.8	17.7	18.8	13.4	24.4
F**inancial literacy**	6.0	17.1	16.7	31.4	28.7
**numeracy skills related to insurance**	51.5	10.5	17.7	6.5	13.8
**Total HIL**	11.5	25.3	35.6	18.1	9.5

**Table 4:** Mean and Standard Deviation of Total HIL Score by Province.

**Province**	**Conceptual Knowledge (Mean ± SD) (%)**	**Attitudes towards Health Insurance (Mean ± SD) (%)**	**Insurance Information Seeking** **(Mean ± SD) (%)**	**Utilization of Health Insurance** **(Mean ± SD) (%)**	**Digital literacy** **(Mean ± SD) (%)**	**Financial literacy (Mean ± SD) (%)**	**numeracy skills related to insurance** **(Mean ± SD) (%)**	**Total HIL** **Mean ± SD**
East Azerbaijan	51.41 ± 23.19	73.59 ± 28.23	58.11 ± 26.10	42.30 ± 27.71	56.41 ± 30.73	74.03 ± 24.56	47.43 ± 36.38	55.15 ± 19.82
West Azerbaijan	56.21 ± 25.50	78.91 ± 22.94	60.36 ± 26.30	34.32 ± 23.69	52.25 ± 30.97	65.20 ± 28.73	35.58 ± 35.39	53.32 ± 18.86
Ardabil	67.63 ± 29.21	86.38 ± 22.44	66.66 ± 24.39	53.88 ± 24.48	66.20 ± 30.46	79.86 ± 22.81	48.14 ± 35.36	66.09 ± 20.95
Isfahan	49.02 ± 26.83	77.59 ± 26.05	58.11 ± 26.10	42.30 ± 27.71	56.41 ± 30.73	74.03 ± 24.56	47.43 ± 36.38	55.15 ± 19.82
Alborz	49.34 ± 26.67	78.42 ± 22.39	53.50 ± 22.49	25.86 ± 23.91	47.58 ± 30.51	59.37 ± 24.80	24.78 ± 32.48	45.84 ± 18.17
Ilam	63.59 ± 28.37	84.68 ± 21.09	61.97 ± 25.65	40.15 ± 32.29	51.56 ± 38.88	64.06 ± 28.88	38.54 ± 32.91	57.54 ± 24.04
Bushehr	58.48 ± 20.69	78.69 ± 19.37	58.33 ± 28.53	35.86 ± 21.88	51.44 ± 31.34	64.13 ± 25.92	25.36 ± 32.12	53.02 ± 19.26
Tehran	53.54 ± 25.90	80.08 ± 21.35	59.90 ± 26.01	29.86 ± 23.62	50.21 ± 32.47	63.58 ± 24.28	31.18 ± 31.94	50.53 ± 18.55
Chaharmahal and Bakhtiari	52.22 ± 28.19	77.5 ± 21.29	62.96 ± 28.20	35.00 ± 29.05	46.75 ± 27.83	65.27 ± 23.73	36.57 ± 34.69	51.61 ± 22.83
South Khorasan	50.00 ± 28.98	81.61 ± 21.30	59.67 ± 25.64	22.25 ± 17.69	51.07 ± 33.59	61.25 ± 25.92	21.50 ± 26.24	46.95 ± 16.60
Razavi Khorasan	48.55 ± 26.86	77.03 ± 23.15	52.59 ± 29.46	26.11 ± 23.83	43.45 ± 30.61	59.32 ± 25.77	26.41 ± 32.66	46.05 ± 20.26
North Khorasan	53.78 ± 24.27	87.87 ± 13.86	57.07 ± 21.96	29.09 ± 20.67	87.87 ± 13.86	66.28 ± 22.20	31.81 ± 32.63	51.88 ± 15.77
Khuzestan	47.92 ± 28.06	84.14 ± 17.46	56.91 ± 27.31	34.51 ± 28.27	48.37 ± 28.06	63.75 ± 23.64	34.55 ± 37.52	50.91 ± 21.14
Zanjan	54.90 ± 29.24	78.26 ± 26.62	61.53 ± 26.20	36.63 ± 28.62	50.00 ± 35.39	63.94 ± 28.51	37.17 ± 34.71	53.49 ± 22.40
Semnan	45.68 ± 29.02	77.95 ± 26.37	55.87 ± 25.57	23.86 ± 18.92	37.50 ± 29.88	55.81 ± 26.21	17.04 ± 24.76	43.56 ± 18.94
Sistan and Baluchestan	58.05 ± 28.55	78.33 ± 27.27	66.20 ± 27.03	46.38 ± 29.74	51.85 ± 35.64	62.50 ± 32.08	37.03 ± 35.49	57.31 ± 23.94
Fars	52.02 ± 26.14	78.60 ± 22.68	55.64 ± 27.29	33.42 ± 28.39	50.00 ± 32.79	66.77 ± 24.99	28.90 ± 35.17	50.77 ± 21.15
Qazvin	56.90 ± 28.15	74.52 ± 30.37	56.94 ± 27.41	29.40 ± 22.47	50.39 ± 29.09	67.55 ± 26.05	34.52 ± 36.88	51.27 ± 19.23
Qom	55.78 ± 26.25	85.88 ± 18.67	56.86 ± 24.07	36.86 ± 31.85	52.28 ± 31.27	72.54 ± 22.64	34.64 ± 34.93	54.83 ± 20.67
Kurdistan	45.00 ± 30.28	69.23 ± 29.32	44.87 ± 31.21	29.92 ± 20.41	43.16 ± 28.28	58.33 ± 33	18.37 ± 25.30	42.74 ± 20.85
Kerman	51.38 ± 24.88	85.83 ± 19.03	52.77 ± 30.73	36.25 ± 27.42	52.77 ± 30.73	62.84 ± 25.61	40.27 ± 35.93	53.62 ± 20.67
Kermanshah	59.76 ± 27.53	78.14 ± 26.83	59.88 ± 27.71	36.51 ± 25.85	57.75 ± 30.93	69.64 ± 24.86	36.04 ± 35.43	55.51 ± 20.34
Kohgiluyeh and Boyer-Ahmad	67.63 ± 26.00	91.05 ± 11.96	67.10 ± 24.91	35.00 ± 31.84	51.74 ± 32.81	68.42 ± 26.14	33.33 ± 38.49	58.85 ± 19.94
Golestan	44.26 ± 26.18	76.09 ± 26.54	50.81 ± 27.43	29.39 ± 20.56	42.68 ± 31.19	64.02 ± 27.69	34.95 ± 37.97	46.60 ± 19.57
Gilan	52.87 ± 23.82	73.75 ± 27.14	55.62 ± 26.97	30.87 ± 25.56	47.50 ± 35.91	63.43 ± 23.74	29.58 ± 31.68	49.39 ± 19.58
Lorestan	51.63 ± 31.39	57.82 ± 36.99	54.89 ± 32.42	45.21 ± 32.45	54.34 ± 32.66	58.15 ± 29.83	40.57 ± 36.96	51.32 ± 26.71
Mazandaran	48.40 ± 28.50	80.90 ± 22.90	55.30 ± 31.18	30.45 ± 26.43	42.42 ± 30.17	58.52 ± 25.25	38.63 ± 33.09	48.83 ± 31.18
Markazi	56.25 ± 25.22	82.5 ± 23.46	51.85 ± 29.95	28.05 ± 20.11	45.37 ± 29.71	62.15 ± 25.44	30.55 ± 35.74	49.83 ± 18.86
Hormozgan	51.45 ± 25.97	85.41 ± 19.99	66.31 ± 27.08	47.08 ± 29.00	49.30 ± 30.88	69.27 ± 27.57	36.11 ± 34.63	57.16 ± 21.26
Hamedan	58.37 ± 24.47	77.5 ± 26.18	58.33 ± 28.92	39.00 ± 25.24	54.58 ± 34.38	66.56 ± 23.74	34.16 ± 31.79	54.72 ± 20.11
Yazd	45.97 ± 28.72	76.30 ± 22.83	56.70 ± 25.67	30.32 ± 23.90	44.56 ± 29.40	54.34 ± 25.98	32.60 ± 31.61	47.16 ± 19.36

The results of the correlation analysis revealed a significant relationship between age and overall health insurance literacy (P < 0.0001), knowledge (P < 0.0001), attitude toward health insurance (P < 0.0001), numeracy skills(P < 0.0001) information-seeking behavior (P < 0.01) and financial literacy(P < 0.0001).

### 2.3 Multiple linear regression analysis

The stepwise regression model was statistically significant (F (3,1618) = 44.99, p < 0.001), explaining 7.7 % of the variance in HIL scores (R^2^ = 0.077, Adjusted R^2^ = 0.075). Higher education (B = 6.71, 95 % CI: 4.61–8.81, p < 0.001), employment (B = 2.73, 95 % CI: 1.78–3.69, p < 0.001), and older age (B = 0.08, 95 % CI: 0.007–0.162, p = 0.03) were significantly associated with greater HIL. Among these, education was the strongest predictor (β = 0.160). Multicollinearity was not a concern (all VIF < 1.3), and residual diagnostics confirmed that the assumptions of linear regression were satisfied (Table 5).

**Table 5:** Multiple linear regression Analysis of the Relationship between HIL Score and Demographic Variables.

**Variable**	**B (Unstandardized)**	**SE**	**β (Standardized)**	**95 % Confidence Interval**	**P-value**
Education	6.71	1.07	0.160	[4.61, 8.81]	< 0.0001
Occupation	2.73	0.48	0.152	[1.78, 3.69]	< 0.0001
Age	0.08	0.03	0.05	[0.007, 0.162]	0.03

**R^2^:0/077**.

**60**–**70**.

## 3 Discussion

The findings revealed a mean HIL score of 50.85 ± 20.41, indicating a moderate level of literacy among participants. This result aligns with prior research; for instance, a 2024 cross-sectional study in Tehran similarly reported that most respondents exhibited the moderate to low levels of insurance literacy [36]. In contrast, a 2020 study in Ilam found that 74 % of participants demonstrated high insurance literacy, suggesting regional disparities potentially attributable to differences in local insurance policies, public awareness, and access to insurance-related information[34].

International comparisons further underscore how the national insurance systems could influence HIL. A study (2019) in the United States reported that 51 % of participants had inadequate insurance literacy, and 48 % lacked confidence in navigating their insurance to access care[41]. Even in countries with well-developed insurance systems, such deficiencies remain. In contrast, a study by Holst et al. (2022) in the Netherlands reported a mean HIL score of 55.14, which is relatively higher than that in both Iran and the United States[42]. This suggests that robust insurance frameworks and comprehensive public education in developed nations may foster better literacy outcomes.

Evidence consistently shows that health insurance literacy directly influences healthcare utilization. Individuals with higher HIL are more likely to engage in preventive care and primary health services[25], as they are better equipped to make informed decisions and seek timely medical attention. On the other hand, low HIL is associated with delays in receiving essential care and increased healthcare costs. A national study (2018) in United States revealed that individuals with higher insurance knowledge were less likely to experience financial hardship from unexpected medical expenses and managed their insurance coverage more effectively[43]. Enhancing public awareness of health insurance, therefore, can contribute to lowering overall healthcare costs and improving public health outcomes.

The findings of the study indicated that educational attainment was significantly associated with the level of Health Insurance Literacy (HIL). Specifically, individuals with a bachelor’s degree or higher scored higher across multiple dimensions of health insurance literacy compared to those with a high school diploma or lower. This relationship can be attributed to the direct role of education in enhancing cognitive skills, analytical abilities, and the capacity to comprehend complex insurance-related texts and concepts. Individuals with higher levels of education typically perform better in understanding their insurance rights, comparing different plans, tracking coverage, and utilizing services effectively. Conversely, those with lower levels of education may face challenges in understanding insurance concepts, assessing the credibility of received information, or navigating insurance services—factors which render them more vulnerable to being deprived of their insurance rights. This educational gap manifests in terms of not only awareness and access to information but also attitudes toward, and trust in, insurance providers. Consistent with prior studies in both Iran and abroad, individuals with higher levels of education demonstrated better understanding of insurance concepts and more effective use of insurance services[34]. Research from the Netherlands and the U.S. shows that individuals with lower education levels often struggle not only with comprehension but also with selecting and utilizing insurance plans, underscoring the need for targeted educational interventions for less-educated populations[42].

The findings of the study indicated that there was a significant relationship between age and the level of Health Insurance Literacy (HIL). Older individuals—particularly those in middle and late adulthood—scored higher on the HIL index compared to younger age groups. This positive association can be attributed to increased experience, more frequent interaction with the healthcare system, and greater personal and familial responsibilities in later stages of life—factors that enable older adults to develop a more accurate understanding of health insurance structures, healthcare service use, and their rights related to insurance. Additionally, due to their recurrent encounters with illness, their regular medical visits, and their necessity to use insurance, older individuals gradually acquire more advanced skills in navigating the insurance system. In contrast, younger individuals—especially those under the age of 30—owing to relatively better health, lack of immediate need for medical services, and limited awareness of how health insurance functions, often demonstrate lower levels of insurance literacy. Similar age-related trends have been reported in Iran, Switzerland, and the U.S., with younger individuals consistently showing lower literacy[19,34,43,44]. One possible explanation, grounded Kruger & Dunning (1999) theory, is that younger individuals may underestimate their competence in performing specific tasks due to limited practical experience [45]. In the context of health insurance, this may result in reduced self-confidence and literacy levels among youth.

Given these findings, there is a clear need for educational programs targeting young people, aimed at familiarizing them with insurance concepts, plan selection, and optimal use of coverage. Opportunities for practical engagement and decision-making at earlier ages could also contribute to improving literacy in this demographic.

The results of the study revealed that employment status is one of the most significant and influential factors affecting the level of Health Insurance Literacy (HIL). Employed individuals—particularly government employees and those working in the formal sector—scored higher across various dimensions of HIL compared to unemployed individuals, homemakers, and those in informal occupations. This disparity can be attributed to several key factors. First, employed individuals are typically covered by organizational insurance and have access to insurance-related information, training, and procedures through their workplace. Second, the structured nature of formal employment encourages more active use of insurance benefits, while daily interactions with healthcare, administrative, and financial systems enhance their functional knowledge of insurance. In contrast, unemployed individuals or those engaged in informal jobs (such as street vendors or day laborers) not only have limited access to informational resources, but they are also often inadequately insured or unable to effectively utilize available services due to bureaucratic complexities.

The geographical analysis of the present study revealed considerable inter-provincial disparities in Health Insurance Literacy (HIL) across Iran. Ardabil Province reported the highest average HIL score, while Kurdistan Province had the lowest. These regional differences are likely influenced by a combination of economic conditions, cultural norms, and policy environments. Provinces with stronger socioeconomic infrastructure and broader access to health-related information resources generally demonstrated higher levels of insurance literacy. In contrast, more disadvantaged areas may suffer from limited access to educational and informational tools necessary for understanding and utilizing health insurance systems effectively.

Surprisingly, however, some less developed provinces—such as Ardabil, Kohgiluyeh and Boyer-Ahmad, and Sistan and Baluchestan—exhibited higher HIL scores than more developed provinces like Tehran, Isfahan, and Alborz. For example, Ardabil recorded an average score of 66.17, compared to Tehran’s 50.96. Several methodological and contextual factors may explain this unexpected pattern. First, the study’s online and SMS-based sampling method inherently included individuals with digital access and familiarity with IHIO’s electronic services. This may have excluded vulnerable and less-informed populations, particularly in urban centers, while capturing a more selective and digitally literate subgroup in disadvantaged provinces. As a result, the study may have overestimated HIL levels in certain areas, particularly where sample sizes were small (e.g., Sistan and Baluchestan with only 18 respondents), thus weakening the precision of provincial comparisons.

Second, differences in the demographic composition of respondents across provinces likely contributed to the observed disparities. The study confirmed significant associations between HIL and education, occupation, income, marital status, and type of insurance coverage. Provinces with an overrepresentation of more educated or formally employed individuals—despite overall lower socioeconomic development—may therefore report artificially elevated HIL scores. For instance, individuals insured through the Government Employees Fund consistently scored higher than those in other funds, and the distribution of fund types is not uniform across provinces.

Beyond methodological considerations, however, sociocultural mechanisms may also offer insight. Evidence indicates that community cohesion, collective trust in public institutions, and cultural norms surrounding healthcare access can shape insurance-related behaviors independent of socioeconomic status [13,46]. In Iran, disadvantaged provinces have historically been prioritized by targeted insurance expansion policies, which may have enhanced awareness and engagement with insurance services in these regions. Similar findings in other low- and middle-income countries suggest that differences in HIL are mediated not only by wealth or education but also by contextual factors such as information-sharing practices, local expectations, and policy environments [14,47].

Taken together, these findings highlight the complex interplay between structural disadvantage, demographic composition, and sociocultural context in shaping health insurance literacy. While methodological limitations caution against overinterpreting provincial differences, the evidence underscores the need for mixed-methods research that combines quantitative measurement with qualitative inquiry to better capture underlying mechanisms. Such approaches would enable a deeper understanding of why certain disadvantaged provinces outperform expectations and would provide more nuanced evidence for designing culturally sensitive, equity-focused interventions to improve HIL nationwide.

Another noteworthy factor is the type of insurance coverage. The study found that individuals insured through the Government Employees Insurance Fund had significantly higher levels of HIL than those affiliated with other insurance funds. Given the unequal distribution of insurance types across provinces, this imbalance may have further influenced the provincial HIL averages.

Taken together, these findings highlight the complex interplay between structural, demographic, and methodological factors in shaping health insurance literacy outcomes. Policymakers and health system stakeholders must therefore consider both individual-level determinants and systemic disparities when designing interventions aimed at improving insurance literacy, particularly in underserved and socioeconomically disadvantaged regions.

A key strength of this study lies in its use of a large, nationally representative sample and a validated, multidimensional assessment tool, enhancing the generalizability and credibility of its findings. This research fills a significant gap in Iranian and LMIC literature by providing empirical data on HIL across diverse demographic groups. Its novelty stems not only from the breadth of its dataset but also from its potential to inform scalable, culturally adapted, and evidence-based interventions.

Nevertheless, several limitations must be acknowledged. First, the cross-sectional design limits causal inferences between HIL and demographic factors. Second, the online survey method may have introduced self-selection bias, potentially excluding individuals with limited internet access or digital literacy. To minimize response bias, the survey was administered anonymously and incorporated logic checks to detect and exclude inconsistent responses. Additionally, the HIL instrument combined objective numeracy and knowledge-based items with self-assessment components to enhance construct validity.

## 4 Conclusion

This study, utilizing a nationally representative sample and a validated assessment tool, provides a comprehensive overview of the state of Health Insurance Literacy (HIL) among beneficiaries of the Iranian Health Insurance Organization. The findings indicate that the overall level of HIL in Iran is moderate and significantly influenced by factors such as age, gender, education, employment status, income level, type of insurance fund, and geographical location. Specifically, the men, individuals with higher education, formal employment, and higher income showed higher levels of HIL. Moreover, substantial inter-provincial disparities were observed, likely reflecting a combination of demographic, structural, and cultural differences.

The results suggest that enhancing HIL can lead to more informed utilization of insurance services, reduced healthcare costs, and improved health outcomes. Given the direct link between HIL and equitable access to healthcare, it is crucial to design and implement the targeted educational and informational interventions—especially for vulnerable groups such as youth, homemakers, low-income individuals, and residents of underserved regions. Revising insurance structures, expanding workplace and clinical training programs, and strengthening digital infrastructure may also contribute to improving HIL nationwide.

Ultimately, this research fills a critical gap by offering robust empirical evidence that can inform the development of national policies and scalable, culturally adapted interventions aimed at improving health insurance literacy and reducing disparities in service utilization in Iran and other low- and middle-income countries (LMICs). Probing over the underlying reasons for the lower HIL at the different provinces could be envisaged as the future avenue of HIL research.

### 4.1 Policy implications

These findings highlight the importance of targeted interventions: (1) developing educational modules on insurance concepts for younger and less-educated populations, (2) leveraging workplaces as channels for insurance training, and (3) designing community-based outreach programs in underserved regions. Such initiatives could enhance insurance literacy, reduce disparities in healthcare utilization, and ultimately improve financial protection in the Iranian healthcare system.

## CRediT authorship contribution statement

**Zahra Asadi-Piri:** Conceptualization, Writing – original draft. **Foroozan Abdollahi-pour:** Data collection and manuscript drafting. **Namam Ali Azadi:** Statistical analysis and data interpretation. **Rajabali Daroudi:** Manuscript revision. **Ebrahim Jaafaripooyan:** Conceptualization, Review & editing, Final Supervision.

## Declaration of competing interest

The authors declare that they have no known competing financial interests or personal relationships that could have appeared to influence the work reported in this paper.
